# p66Shc Aging Protein in Control of Fibroblasts Cell Fate

**DOI:** 10.3390/ijms12085373

**Published:** 2011-08-22

**Authors:** Jan M. Suski, Agnieszka Karkucinska-Wieckowska, Magdalena Lebiedzinska, Carlotta Giorgi, Joanna Szczepanowska, Gyorgy Szabadkai, Jerzy Duszynski, Maciej Pronicki, Paolo Pinton, Mariusz R. Wieckowski

**Affiliations:** 1 Department of Biochemistry, Nencki Institute of Experimental Biology, Warsaw 02-093, Poland; E-Mails: j.suski@nencki.gov.pl (J.M.S.); mlebiedz@nencki.gov.pl (M.L.); j.szczepanowska@nencki.gov.pl (J.S.); jdus@nencki.gov.pl (J.D.); 2 Department of Experimental and Diagnostic Medicine, Section of General Pathology, Interdisciplinary Center for the Study of Inflammation (ICSI) and LTTA Center, University of Ferrara, Ferrara 9-44121, Italy; E-Mails: grgclt@unife.it (C.G.); pnp@unife.it (P.P.); 3 Department of Pathology, The Children’s Memorial Health Institute, Warsaw 04-730, Poland; E-Mails: medycyna.mitochondrialna@czd.pl (A.K.-W.); m.pronicki@czd.pl (M.P.); 4 University College London, Department of Cell and Developmental Biology, Consortium for Mitochondrial Research, London WC1E 6BT, UK; E-Mail: g.szabadkai@ucl.ac.uk

**Keywords:** p66Shc, reactive oxygen species, antioxidant defense, mitochondria

## Abstract

Reactive oxygen species (ROS) are wieldy accepted as one of the main factors of the aging process. These highly reactive compounds modify nucleic acids, proteins and lipids and affect the functionality of mitochondria in the first case and ultimately of the cell. Any agent or genetic modification that affects ROS production and detoxification can be expected to influence longevity. On the other hand, genetic manipulations leading to increased longevity can be expected to involve cellular changes that affect ROS metabolism. The 66-kDa isoform of the growth factor adaptor Shc (p66Shc) has been recognized as a relevant factor to the oxygen radical theory of aging. The most recent data indicate that p66Shc protein regulates life span in mammals and its phosphorylation on serine 36 is important for the initiation of cell death upon oxidative stress. Moreover, there is strong evidence that apart from aging, p66Shc may be implicated in many oxidative stress-associated pathologies, such as diabetes, mitochondrial and neurodegenerative disorders and tumorigenesis. This article summarizes recent knowledge about the role of p66Shc in aging and senescence and how this protein can influence ROS production and detoxification, focusing on studies performed on skin and skin fibroblasts.

## 1. Introduction

A broad body of evidence describes the tissue-specific thresholds of reactive oxygen species (ROS) which, if surpassed, exert oxidative stress. Harman’s free radical theory of aging, proposes that an uncontrolled increase of ROS results in oxidative damage of fundamental cellular components such as proteins, phospholipids and DNA [1]. Accumulation of such damages has a negative impact on the functions of tissues and organs, leading to aging and various pathologies. Both, oxidative and genetic-related defects of the mitochondrial respiratory chain, age-dependent decrease of the antioxidant defense system efficiency, as well as the activity of certain proteins (such as p66Shc), make a relevant contribution to enhanced intracellular ROS production. Investigation of these mechanisms, especially the pivotal properties of p66Shc, permits a better understanding of aging. Here, we attempt to systematize the existing knowledge governing the activity of p66Shc in processes of aging and the emergence of various pathologies associated with oxidative stress.

## 2. Age-Related Dysfunctions of the Mitochondrial Respiratory Chain and Antioxidant Defense

**Figure 1.**
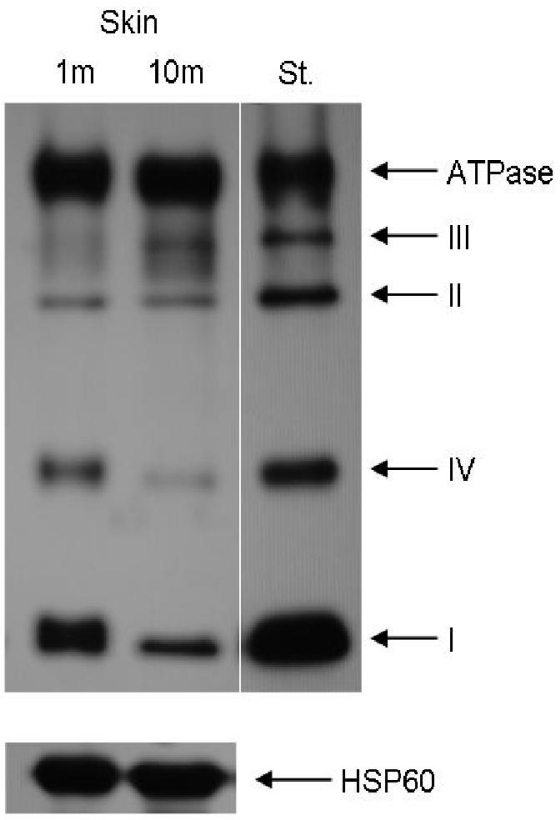
Age-dependent changes in the expression of respiratory chain subunits in mouse skin. Expression profile of respiratory chain subunits in skin from 1-month-old and 10-month-old mouse; St.—standard (mitochondria isolated from mouse heart); HSP60-marker of mitochondrial content in the sample. Antibodies and experimental protocols were as previously described [12,13].

Several lines of evidence indicate that in different tissues the activity of some mitochondrial respiratory complexes declines with age. In rodents the age-dependent decrease in electron transfer chain (ETC) activity in a variety of tissues (e.g., brain, kidney, heart and liver) refers mainly to complexes I and IV of the respiratory chain. Complex II and III remain practically unaffected during aging [2–7]. In 1994 Boffoli *et al.* [2] demonstrated that a decrease in the activity of particular respiratory chain complexes can be correlated with the reduction of their content in mitochondria as illustrated in Figure 1. This phenomenon was confirmed by a number of authors (reviewed by Van Remmen *et al.* [8]).

Generation of ROS due to leakage of electrons from the ETC is an inherent characteristic of mitochondrial respiration. ROS, in accordance to Harman’s free radical theory of aging, are the main contributor in this process [1]. An inhibition of the respiration rate and corresponding higher reduction of the respiratory chain components, due to decreased respiratory chain complexes content elevates electron leakage and ROS formation. In accordance with the above mentioned observations, mitochondrial ATP synthesis has been reported to decrease with age in different human tissues [9,10] and skin fibroblasts [11].

**Figure 2.**
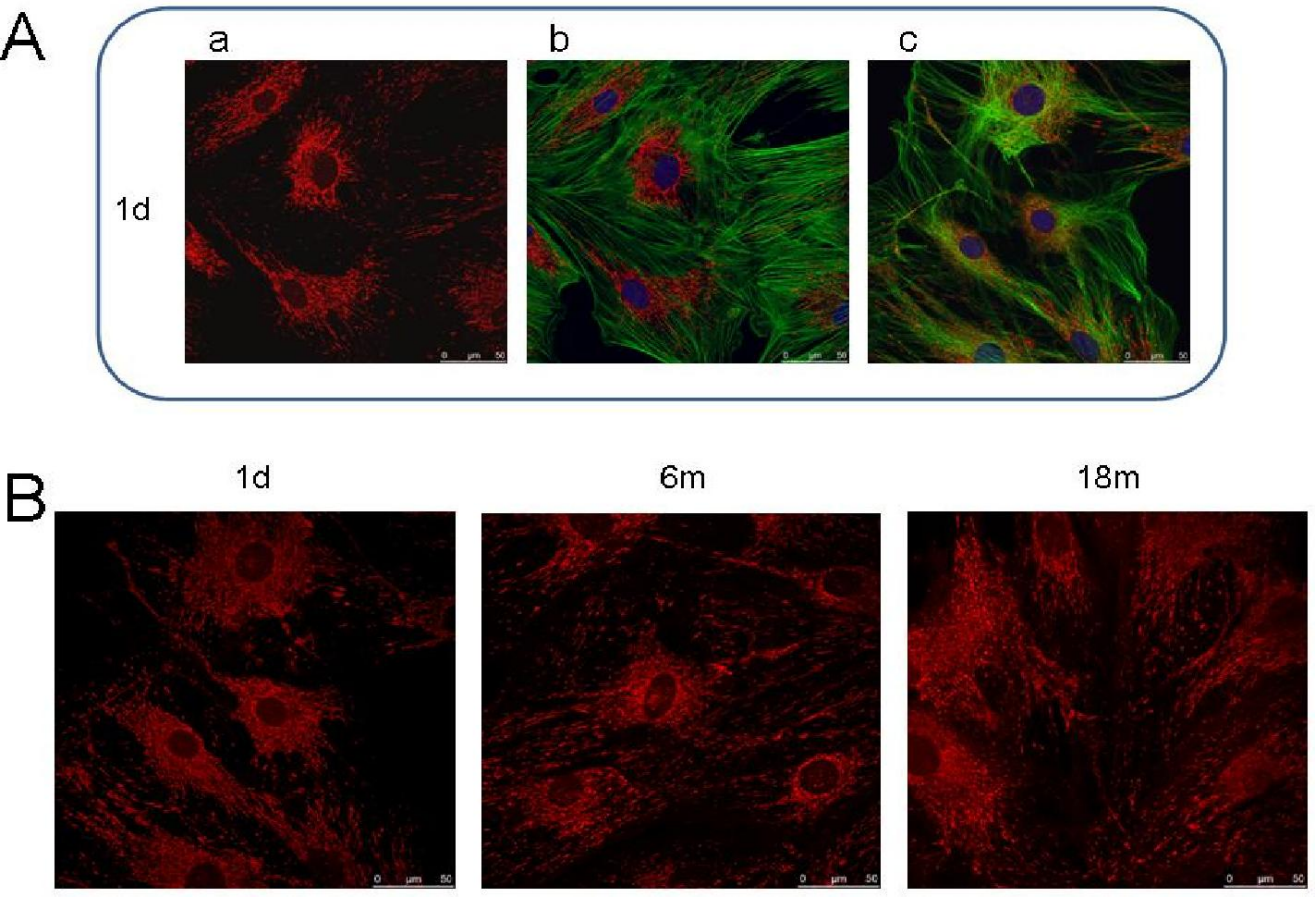
Age-dependent changes in mitochondrial structure of mice fibroblasts. (**A**) Mitochondrial structure (**a**), actin filaments (**b**) and microtubules (**c**) organization in fibroblasts from 1-day-old (**1 d**) mice; and (**B**) Age-dependent changes in mitochondrial structure of mice fibroblasts from 1-day-old (**1 d**), 6-month-old (**6 m**) and 18-month-old (**18 m**) mice. Mitochondrial structure, actin filaments and microtubules organization was visualized as previously described [15].

In a study on primary human fibroblasts derived from subjects of different age groups ranging from 20 weeks fetal to 103 years, Greco *et al.* characterized several mitochondrial processes underlying the capacity of oxidative phosphorylation (OXPHOS) during aging. The following mitochondrial parameters were monitored: mitochondrial protein synthesis, the respiratory rate and a coupling of the oxydative phosphorylation. The authors observed a very significant reduction in the ratio of uncoupled to oligomycin inhibited endogenous respiration in aged fibroblasts which pointed to a decrease with donor’s age in the control of respiration by the mitochondrial membrane potential. The authors also describe a decrease in the respiratory control ratio with the subject’s age. Greco and co-workers also underlined the plausibility of a hypothesis in which ROS play a key role in such deterioration by affecting both mtDNA and nDNA and finally leading to insufficient mitochondrial protein levels as well as mitochondrial lipid peroxidation [11]. Age-dependent alterations also affect mitochondrial morphology. Mitochondria in cells of aged individuals (e.g., mice) have altered intracellular organization and increased volume as illustrated in Figure 2 [14].

### 2.1. Antioxidant Defense and Aging

Though the intracellular free radical production brings a potent danger to the cell, contributing to various pathologies and aging process, cells defend themselves with the multifactorial antioxidative system which includes scavenging enzymes and compounds. In the mitochondrial matrix, manganese superoxide dismutase (MnSOD or SOD2) converts the superoxide anion to hydrogen peroxide, which is then detoxified in the cytosol by catalase to water molecule. The mitochondrial matrix also contains glutathione peroxidise (GPX) and two peroxiredoxins (Prx 3 and 5) that eliminate hydrogen peroxide (H_2_O_2_). The mitochondrial intermembrane space and the cytosol contain the zinc-copper superoxide dismutase (Zn-Cu SOD or SOD1) whose activation depends on the intracellular redox state [16]. An important role in redox homeostasis is played by the glutathione system. The GSH/GSSG balance is kept by glutathione metabolism enzymes: glutathione peroxidise and glutathione reductase. They both are responsible for keeping the proper balance between oxidized and reduced forms of gluthatione and correspondingly they control the redox level within the cell. Furthermore, they control the level of ROS [17]. It was documented by many investigators that antioxidant enzyme levels change with age. Their decrease together with the increase in ROS level might be harmful to proteins, phospholipids and mtDNA [18].

Cell susceptibility to oxidative damage depends on the antioxidant defense efficiency. Thus in cases of aberrant scavenging ability, cells are more prone to premature aging and carcinogenesis due to oxidative DNA damage. Many animal models with antioxidant enzyme deficiencies show higher susceptibility to prooxidant factors such as toxic agents and radiation [19]. On the other hand, boosting antioxidant defense system can potentially prevent pathological changes and may delay aging. For example in case of long-living Ames dwarf mice, dermal fibroblasts are resistant to mitochondrially generated ROS due to upregulation of stress responding cascades [20]. During maturation and aging cells lose their ability to defend against ROS. These characteristics are also manifested in cell cultures in the late passages, and are referred to as proliferative senescence. It is caused by an accumulation of peroxidation products, decrease of non-enzymatic antioxidants, e.g., Coenzyme Q10, and decline in antioxidant enzymes efficiency. There is a significant loss of mitochondrial function in aging, represented by lower mitochondrial membrane potential, higher superoxide production by the electron transfer chain accompanied by lower ATP production and a more frequent appearance of oxidized proteins. CoQ10 administration to the human skin not only seems to reduce visible age-associated changes but prevents DNA oxidation which was suggested to protect against aging and prevent carcinogenesis [21]. Cells that are particularly exposed to external stress factors are skin fibroblasts. UV radiation (UVA and UVB) seems to be very harmful for them inducing free radical production, which leads to senescence as well as dermatological complications. Such enhanced skin aging process (as a consequence to UV exposure) is called photoaging [22,23].

### 2.2. Antioxidant Supplementation

It has been indicated by many studies that supplementation of cell culture media with antioxidants improves mitochondrial metabolism and other cell functions by decreasing oxidative stress. Various mechanisms have been suggested to underlie such benefits, from simple radical reduction to inducing complex signal cascades responsible for antioxidant defense and repair. Antioxidant supplementation may be particularly important in cases of age-associated loss of antioxidant capacity. Cells growing in the presence of compounds possessing antioxidant capacity, such as vitamin E, display prolonged proliferative lifespan and reduced senescence [24,25]. Effective protection against photoaging can be provided by vitamin C and E supplementation. Since dietary intake of these compounds may be not sufficient, additional topical vitamin C application from cosmetics maintains its high level in dermal fibroblasts resulting in DNA protection and an increase in collagen synthesis [24,25]. Aging fibroblasts are characterized by the decline in respiratory function and partial uncoupling. Such defects in oxidative phosphorylation result in insufficient energy supply which affects the proliferative potential of a cell [26]. In such a case, ascorbate supplementation of human fibroblasts can restore enzymatic function of the mitochondrial respiratory chain complexes which results in a delay of aging associated with mitochondrial dysfunction [27]. Nowadays, a number of natural compounds from the group of polyphenols and isoflavones are being tested as antioxidants. Some of them, apart from scavenging reactive oxygen species, also improve antioxidant defense, mitochondrial energy metabolism and retard aging process [28]. For example even short incubation (24 h) with the Epigallocatechin-3-gallate (EGCG), from green tea extract, increases expression of main antioxidant enzymes (catalase, SOD1, SOD2 and glutathione peroxidase). Longer treatment results in significant decrease of intracellular oxidative stress and higher DNA protection [29]. Sesamol, a polyphenolic compound, which is a very potent agent against UVB-induced oxidative stress in dermal fibroblasts, represents similar characteristics. It not only increases antioxidant enzyme levels but also decreases the level of lipid peroxides which are markers of cell damage during aging process [30]. Recent studies with antioxidants mitoQ and SkQ which are targeting specifically mitochondria seems promising [31]. SkQ works in very low concentrations, efficiently preventing mitochondrial cardiolipin oxidation and H_2_O_2_-induced apoptosis in cultured human fibroblasts. Moreover SkQ treatment decreases the occurrence of spontaneous apoptosis in fibroblasts from old individuals to the level observed in cells from young animals [31].

p66Shc is a protein that partially participates in above described age (and ROS)-dependent mitochondrial dysfunctions and cellular pathologies. Its involvement in the cellular response to oxidative stress, resulting in higher/additional ROS production by mitochondria and the ability to influence the intracellular level of antioxidant enzymes was demonstrated in primary cultures of mouse skin fibroblasts [32,33] as well as in fibroblasts derived from patients harboring different mitochondrial dysfunctions [12].

## 3. p66Shc—The Oxidative Stress and Longevity

In 1999 the group of Pelicci [33] published an observation pointing to the role of p66Shc protein in oxidative stress and aging. The authors proposed that the mammalian life span can be controlled by the p66Shc protein and importantly, this effect is due to the regulation of the cellular response to the oxidative stress. These intriguing studies on the transgenic mice lacking p66Shc showed that mice that lack the gene appear to have a 30% increase in life span (compared to mice with a wild type phenotype) without pathological consequences such as increase of tumor frequency and endocrinological abnormalities. Studies of Tomilov *et al.* [34] showed that decreased superoxide production in macrophages of p66Shc^−/−^ mice may result in a lower inflammation status and can protect animals from oxidative injury. Taken together these results partly explain the phenomena of the longevity of the p66Shc lacking mice [34]. However, the lack of p66Shc (which is a negative regulator of p46Shc/p52Shc-mediated Ras signaling) results also in an enhancement of lymphocyte proliferation and of immune responses to the antigen exposure. These changes can correspond to development of autoimmune diseases in p66Shc knock-out mice [35].

## 4. p66Shc—One Protein Two Functions

The p66Shc protein is a growth factor adaptor protein which together with p52Shc and p46Shc belongs to the ShcA family. All ShcA proteins have similar domain structure and contain three functionally identical domains: the carboxy terminal Src homology 2 (SH2) domain, central proline-rich domain (CH1), and the *N*-terminal phosphotyrosine-binding domain (PTB). The structure and function of the individual domains has been extensively described in [36]. p66Shc differs from p56Shc and p46Shc by the presence of an additional *N*-terminal proline-rich collagen-homology domain (CH2), which contains a serine phosphorylation site (Ser36) important for the “proapoptotic” properties of p66Shc [36,37]. Moreover, p66Shc contains a functional region (CCB) responsible for interaction with cytochrome c [38]. Under physiological condition p66Shc is involved in the signal transduction (modulation) from, e.g., epidermal growth factor (EGF) receptor to the nucleus. When phosphorylated at tyrosine residues, p52Shc and p46Shc have been found to bind to the Grb2/SOS complex—an activator of the Ras protein. p66Shc can be phosphorylated at tyrosine and it can also interact with Grb2, but there is no evidence that in this way it can activate the Ras signaling pathway. Therefore, p66Shc competes with p52Shc to bind Grb2, which suggests that p66Shc can be a dominant negative regulator of the Ras-mediated signaling pathway [39]. Under oxidative stress (UV exposure or H_2_O_2_ treatment) Ser36 of the p66Shc protein is phosphorylated by one the serine-threonine kinases, protein kinase Cβ (PKCβ), which triggers the signaling pathway leading to apoptosis. This cascade of events has been described by us [32] and consists of four important steps. Briefly: Step 1—in response to the oxidative stress p66Shc is phosphorylated at Ser36. Step 2—phosphorylated p66Shc is isomerised by a prolyl isomerase Pin1. Step 3—isomerised, phosphorylated at Ser36 p66Shc is dephosphorylated by phosphatase A2 (PP2A). Step 4—dephosphorylated p66Shc is finally translocated to the mitochondria where it participates in ROS production [38].

It is still unclear whether p66Shc is translocated across the outer mitochondrial membrane and resides in the mitochondrial intermembrane space where it can interact with cytochrome c [38] or it binds to the outer mitochondrial membrane from the cytosolic side. Recently, our group discovered that p66Shc is also present in plasma membrane-associated membrane (PAM) and in mitochondria-associated membrane (MAM) fractions [13,40,41] and that the level of p66Shc in these fractions changes in an age-dependent manner. MAM fraction isolated from livers of old animals contained more p66Shc than MAM isolated from 1-month-old mice. Moreover, increased levels of p66Shc in crude liver mitochondria (containing MAM) of old mice, correlated positively with enhanced H_2_O_2_ production measured in mitochondrial preparation [13]. However, regardless of the fact of how p66Shc interacts/enters mitochondria, it appears to modulate mitochondrial metabolism and participate in ROS production [42].

## 5. A New Job for p66Shc: Its Role in Adipocyte Metabolism

The deletion of p66Shc has been found to protect mice from a number of degenerative diseases based on cell loss [43]. Moreover, the effect of p66Shc deficiency on atherogenesis has been studied, especially after challenge with a high fat diet, in mice [44]. Major decreases of atherogenesis were observed in p66Shc^−/−^ mice *vs.* controls fed a high-fat diet, and reduced expression of oxidation-specific epitopes was observed in their arterial walls, and reduced vascular apoptosis was observed. Furthermore, plasma isoprostanes (a marker of lipid oxidative damage) were decreased both in unchallenged and fat-challenged p66Shc^−/−^ mice [44].

Apart from this pathological function of p66Shc, this protein also functions in the regulation of insulin signaling and adipocyte metabolism [45]. In fat cells, p66Shc potentiates insulin signaling and triglyceride accumulation. Genetic experiments showed that this effect of p66Shc is mediated by its phosphorylation (on serine-36 phosphorylation) and its ability to generate ROS. Notably, the PTEN phosphatase inhibits insulin signaling and is inactivated by oxidation, suggesting that it may function as one of the targets of p66Shc-generated ROS. Thus, p66Shc-generated ROS might be critical regulators of insulin signaling and fat development. Remarkably, p66Shc^−/−^ mice are protected from diet-induced obesity, suggesting that p66Shc regulates diet-associated fat development [45,46].

Furthermore, p66Shc^−/−^ mice have reduced body weight, due to reduced fat mass of both white and brown adipose tissues [45]. This leanness is not explainable by changes in food intake, intestinal absorption of nutrients or locomotor activity. Rather, it may reflect defects in lipogenesis of adipocytes, as suggested by the reduced lipid accumulation of p66Shc^−/−^ adipocytes transplanted into WT recipient mice [45]. However, this interpretation of the mechanisms leading to decreased fat mass in p66Shc^−/−^ mice poses the question of how energy balance is maintained in the absence of p66Shc, and why energy storage is reduced. As p66Shc^−/−^ mice showed increased basal body temperature and increased basal metabolic rate [45], this suggests that increased uncoupled respiration in the fat mitochondria of p66Shc^−/−^ mice leads to increased energy expenditure, which contributes to resistance to body weight gain. Moreover, Ranieri and colleagues showed that p66 deficiency protects against fat accumulation and premature death in lepOb/Ob mice, an established genetic model of obesity and insulin resistance [46].

However, this lean and resistant to diet-induced obesity phenotype of p66Shc^−/−^ mice cannot be solely the result of p66Shc deletion. Indeed, it has been recently demonstrated as p66Shc^−/−^ mice in addition to p66Shc deletion have a fourfold increase in p46Shc expression in white fat. Thus, p46Shc overexpression in fat, rather than p66Shc deletion, could be the likely cause of decreased adiposity and reduced insulin sensitivity in the fat of p66Shc^−/−^ mice [47].

## 6. Studies on p66Shc in Fibroblast Models

Studies performed on fibroblasts derived from mice lacking p66Shc also demonstrated an increased resistance to oxidative stress [32,33] and lower basal ROS production [33]. In contrast to wild type cells, treatment with H_2_O_2_ of p66Shc knock-out fibroblasts did not affect mitochondrial structure and calcium uptake [32]. Accordingly, accumulation of carbonylated proteins in p66Shc^−/−^ fibroblasts (indicating the scale of the oxidative damage of proteins) was significantly lower as compared to wild type cells. The phenomenon of enhanced resistance to oxidative stress of p66Shc^−/−^ fibroblasts was intensively studied by Nemoto and Finkiel [48]. Based on their data, it can be concluded that Ser36-P-p66Shc additionally acts as a negative regulator of antioxidant enzyme synthesis. FoxO proteins are known to be involved in the modulation of energy metabolism, proliferation and cell death. FoxO transcription factors can also regulate the level of SOD2 and catalase. Ser36-P-p66Shc acts negatively on FoxO which results in decreased amounts of both enzymes. Interplay between the proapoptotic protein p53 and phosphorylated p66Shc which inhibits FOXO3a transcriptional activity resulting in decreased level of catalase and SOD2 has been recently described by Pani *et al.* [49]. Apparently, cells lacking p66Shc are more resistant to apoptotic stimuli like H_2_O_2_.

**Figure 3.**
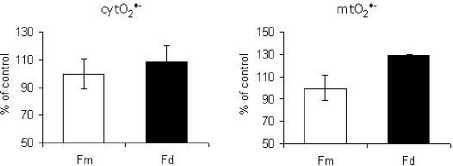
Superoxide production in human dermal fibroblasts. Cytosolic (cytO_2_ ^•−^) and mitochondrial (mtO_2_ ^•−^) superoxide production in neonatal human dermal fibroblasts (NHDF-Neonatal, Cat. n. CC-2509, Lonza) (Fm) and adult human dermal fibroblasts (NHDF-Adult, Cat. n. CC-2511, Lonza) (Fd). Superoxide production was measured as previously described in [12].

In our recent paper we have shown that the level of the Ser36-P-p66Shc is positively correlated with the age of a mouse (in older animals the phosphorylation status of p66Shc is higher) that may partially explain higher superoxide and H_2_O_2_ production observed in old animals [13,50,51]. For example in the skin of adult mice there is more p66Shc and Ser36-P-p66Shc comparing to the material obtained from 1 day old individuals [13]. Interestingly, levels of p66Shc in fibroblasts derived from 18 months old, 6 months old and one day old mice are similar. This result is in variance with the observation of Pandolfi and colleagues that p66Shc is highly expressed in fibroblasts from centenarians [52]. The expression ration between Ser36-P-p66Shc to Actin (1 d = 1.75; 6 m = 1.81; 18 m = 1.89) showed increasing phosphorylation of p66Shc, indicating that the p66Shc pathway was activated. Similarly, the level of p66Shc in neonatal human dermal fibroblasts (NHDF-Neonatal, Cat. n. CC-2509, Lonza) and adult human dermal fibroblasts (NHDF-Adult, Cat. n. CC-2511, Lonza) seems to be expressed at the same level. Both cytosolic and mitochondrial superoxide production in adult fibroblasts have been found increased (see Figure 3), which together with the observed lower level of SOD 2 (as we described in [13]) can indicate the existence of higher oxidative stress in cells from older individuals.

However, NAD/NADH redox state has been found comparable in both cell lines [53]. Decreased level of SOD2 is in an agreement with data already published [54–56] that with age antioxidant defense is less efficient due to decreased activity of antioxidant enzymes like GPX 1, GR and mitochondrial SOD2.

**Figure 4.**
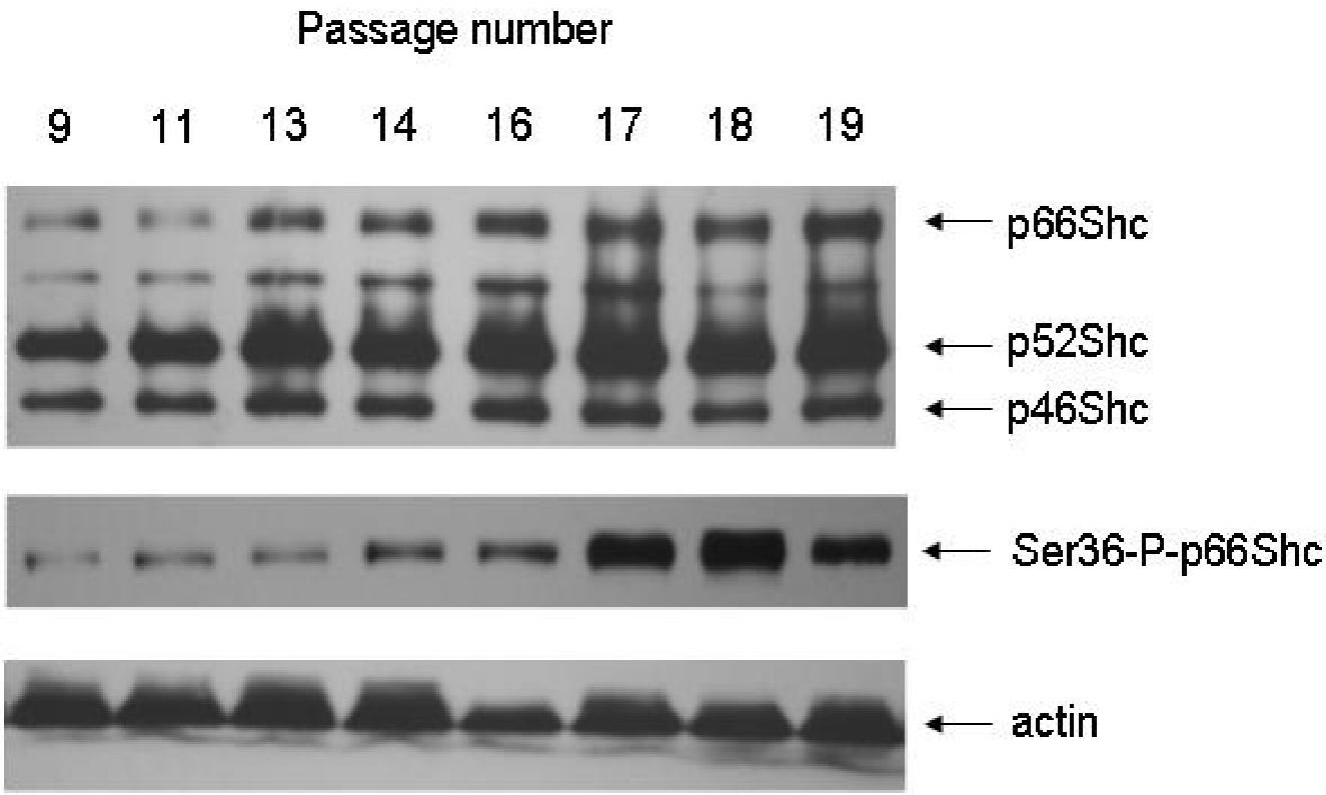
Passage-dependent changes of Shc proteins and Ser36-phosphorylated p66Shc in mouse fibroblasts. Actin was used as loading control. Antibodies and experimental protocols were as previously described in [12].

More straightforward evidence for the participation of the p66Shc protein in mitochondrial function came from replicative senescence as a model of aging. The consequences of excess oxidative stress, caused in part by the altered function of oxidative phosphorylation in replicative senescent human fibroblasts, have been studied by the group of Wei [57–59]. These authors proposed that age-related oxidative stress leads to an increased expression and activation of some nuclear genes that are involved in mitochondrial biogenesis leading in effect to an increase in mitochondrial mass and mtDNA copy number. Such a response to oxidative stress plays a dual role. First, it can increase the supply of energy necessary for such processes as DNA repair and protein synthesis, necessary for cell survival under stress conditions. On the other hand, by further increase in ROS production it can lead to an elevation of oxidative stress and propel the vicious cycle leading to cell aging or death [57–59]. In this model the levels of both p66Shc and Ser36-P-p66Shc significantly increase from passage to passage but the Ser36-P-p66Shc rises even with a faster kinetics. The calculated phosphorylation status of p66Shc (ratio Ser36-P-p66Shc to p66Shc) indicates that more p66Shc is phosphorylated at late passages (see Figure 4).

During aging/senescence, lower efficiency of the antioxidant defense system and gradually accumulated mitochondrial and nuclear DNA damages are the main causes of an increase of intracellular ROS level. This activates PKCβ-dependent phosphorylation of p66Shc which finally not only perturbs mitochondrial structure but also affects their function, e.g., by modifying mitochondrial Ca^2+^ responses [32,42].

## 7. Mitochondrial Ca^2+^ Homeostasis is Compromised in Senescent Cells through a p66Shc Dependent Pathway

**Figure 5.**
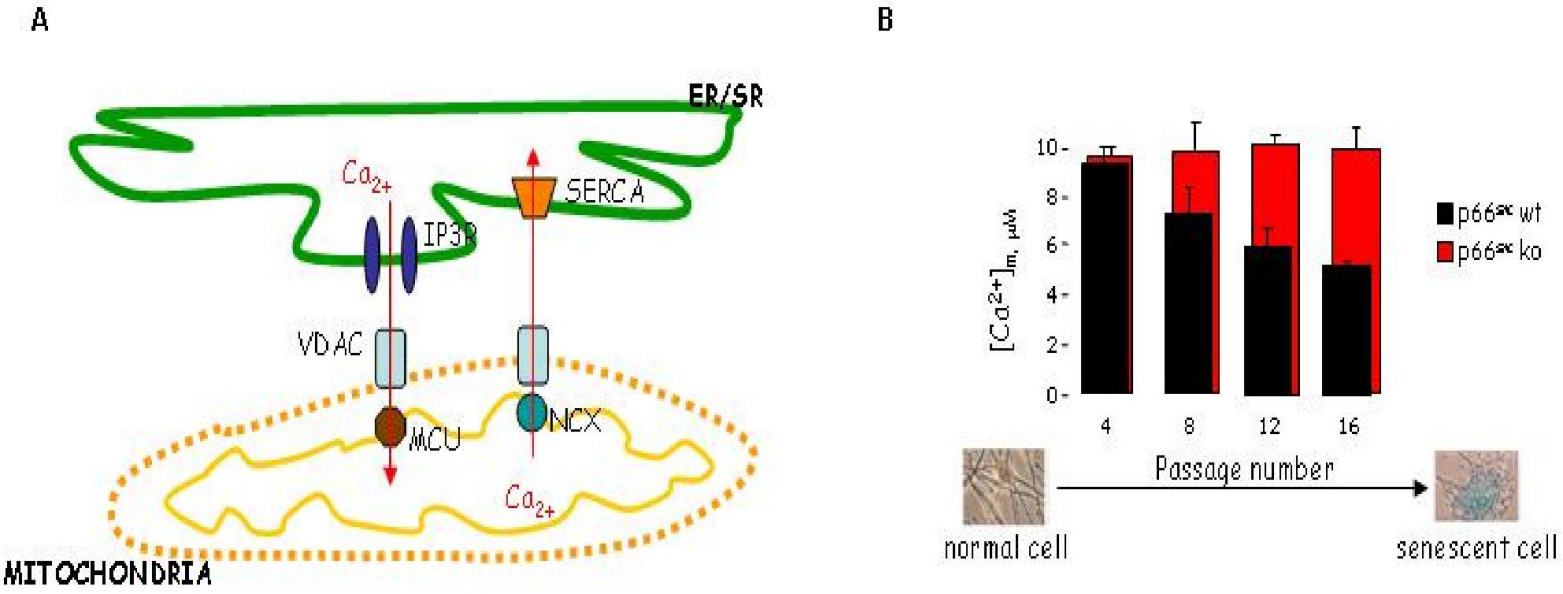
Calcium homeostasis in p66Shc wt and p66Shc^−/−^ cells. (**A**) Mitochondriaendo/sarcopalsmic reticulum (ER/SR) Ca^2+^ crosstalk. MCU—calcium uniporter, NCX—Na^+^/Ca^2+^ exchanger; (**B**) Peak in mitochondrial Ca^2+^ responses after agonist stimulation in wt and p66Shc knockout MEFs (ko) at different passage number. [Ca^2+^]_m_ was measured using the aequorin wt probe as previously described [32].

The electrical gradient across the inner membrane (∼180 mV) represents a strong thermodynamic force in favor of the accumulation of cations like Ca^2+^ [60]. Mitochondrial Ca^2+^ homeostasis has been shown to regulate diverse cellular functions such as cellular ATP production, proliferation, and cell death [61,62]. The processes involved in mitochondrial calcium homeostasis are schematically presented in Figure 5(A).

Stimulation of plasma membrane receptors coupled to a G_q_ protein causes opening of Ca^2+^ channels of the ER membrane (IP3R), and the release of Ca^2+^ from the ER into the cytoplasm. In healthy cells, the inner mitochondrial membrane is nearly impermeable to ions with an exception of Ca^2+^ which transport across the inner mitochondrial membrane depends on the activity of the electrogenic “uniporter” (MCU) of low affinity. Inhibition of the respiratory chain or collapse of the electrical gradient (e.g., by the use of a protonophore) abolishes the capacity of mitochondria to accumulate Ca^2+^. The specific zones of close contact between ER and mitochondria cause that mitochondria are exposed to microdomains of high [Ca^2+^] that largely exceed the values reported in the bulk cytosol and meet the low affinity of the uniporter [63].

In primary cell cultures, such as the mouse embryonic fibroblasts (MEFs) the [Ca^2+^]_m_ responses after agonist stimulation, gradually decreased with time in culture (and thus with the passage number) whereas no alteration was observed in p66Shc^−/−^ MEFs (see Figure 5(B)) [32]. Moreover, inhibition of p66Shc phosphorylation with the use of hispidin, an inhibitor of PKCβ,also abolished passage-dependent decrease of mitochondrial calcium uptake in wild type fibroblasts [32]. These observations clearly link the progressive mitochondrial damage (and the associated reduced mitochondrial responsiveness) observed during the senescence process to the involvement of the ROS sensor p66Shc.

## 8. p66Shc Pathway Is Activated by Intracellular Oxidative Stress—Studies on Fibroblasts from Patients Harboring Mitochondrial Defects

Additional pieces of evidence for the participation of the p66Shc protein in mitochondrial ROS production came from studies using fibroblasts derived from patients harboring mitochondrial defects [12]. Such skin fibroblasts are regularly used in diagnostics as an alternative to muscle biopsy because they can be easily collected (even post mortem), cultured and stored [64–67]. On the other hand, carrying out mitochondrial diagnostics and studies performed in cultured fibroblasts have serious limitations. First, mitochondrial DNA mutations exhibit varying degrees of heteroplasmy, and may be eliminated in the subsequent culture passages. Moreover, certain mtDNA mutations affecting protein synthesis (tRNA mutations) are quickly eliminated from the culture, whereas mutations of the genes of structural subunits, e.g., of complex I (ND1–6) can “survive” in culture. However, it seems that many of the mitochondrial diseases with Mendelian inheritance can be detected and studied in fibroblasts. This is particularly important in children where mutations in nuclear genes predominate in mitochondrial pathologies and can consist up to 80% of all cases. For this reason in our recently published studies, we used fibroblasts derived from patients with various mitochondrial disorders (mitochondrial DNA mutations in ND3 subunit and tRNA leucine, TAZ gene mutation, MEGDEL association) [12].

The signaling pathway involving p66Shc phosphorylation described earlier refers to the situation in which oxidative stress is induced, e.g., by H_2_O_2_ addition to the fibroblasts culture. In such a case p66Shc phosphorylation on Ser36 is caused by extracellular oxidative stress. Our recent studies showed that also intracellular oxidative stress (of mitochondrial origin) activates p66Shc phosphorylation on Ser36. In the case of patients’ fibroblasts (mitochondrial dysfunction results in significantly increased intracellular oxidative stress) phosphorylation of p66Shc on Ser36 was significantly increased. Moreover, we found a substantially decreased level of SOD2 in these cells what additionally confirmed the observation that SOD2 is under control of the p66Shc Ser36 phosphorylation status [12].

## 9. Concluding Remarks

Former studies indicate that, disregarding the source of the oxidative stress, phosphorylation of p66Shc on Ser36 affects mitochondrial metabolism and induces mitochondrial ROS production. This consequently increases intracellular oxidative stress and initiates a vicious circle of p66Shc-dependent ROS production. Completely understanding the role of p66Shc protein in cell physiology and pathology will require further extensive studies that in the future will permit a better understanding of the mechanisms governing processes like aging and various pathologies associated with oxidative stress. Additionally, the assessment of p66Shc phosphorylation changes in mitochondrial patients’ fibroblasts may represent a very promising approach to study to what extent the endogenous ROS overproduction contributes to tissue damage in various genetic mitochondrial defects of known, and especially of unknown, molecular origin. Prospective future therapies of mitochondrial disorders may also be tested by this method.

## Ethics

Presented in the review data from the studies with the use of human fibroblasts were carried out in accordance with the Declaration of Helsinki of the World Medical Association and was approved by the Committee of Bioethics at the Children's Memorial Health Institute. Informed consent was obtained from the parents before any biopsy or molecular analysis was performed. Presented data from the animal studies were conducted in accordance with the Guidelines for the Care and Use of Laboratory Animals prepared by the Polish Academy of Sciences and with approval from Local Ethics Committee.
